# Impact of the COVID-19 pandemic on the incidence and type of infections in hospitalized patients with cirrhosis: a retrospective study

**DOI:** 10.1038/s41598-024-52452-2

**Published:** 2024-02-01

**Authors:** Berta Cuyàs, Anna Huerta, Maria Poca, Edilmar Alvarado-Tapias, Anna Brujats, Eva Román, Carlos Guarner, Àngels Escorsell, German Soriano

**Affiliations:** 1 Department of Gastroenterology, Hospital Santa Creu i Sant Pau, Institut de Recerca Sant Pau, Barcelona, Spain; 2 Department of Medicine, Universitat Autònoma de Barcelona, Barcelona, Spain; 3 Centro de Investigación Biomédica en Red de Enfermedades Hepáticas y Digestivas (CIBERehd), Instituto de Salud Carlos III, Madrid, Spain; 4 Escola Universitària d’Infermeria EUI-Sant Pau, Universitat Autònoma de Barcelona, Barcelona, Spain

**Keywords:** Liver cirrhosis, Bacterial infection, Liver cirrhosis

## Abstract

Infections are a major cause of morbidity and mortality in cirrhosis, especially those caused by multi-drug resistant bacteria. During the COVID-19 pandemic, the incidence and type of infection in these patients may have been influenced by the restrictive measures implemented. We aimed to compare the infections in patients with cirrhosis hospitalized before the COVID-19 pandemic versus those hospitalized during the pandemic. We retrospectively compared infections in patients with cirrhosis hospitalized in the hepatology unit during the pre-pandemic period (3/2019–2/2020) with infections in patients hospitalized during the pandemic (3/2020–2/2021). Baseline characteristics, type of infections, type of bacteria, antimicrobial resistance and mortality were evaluated. There were 251 hospitalizations in 170 patients during the pre-pandemic period and 169 hospitalizations in 114 patients during the pandemic period. One or more infections were identified in 40.6% of hospitalizations during the pre-pandemic period and 43.8% of hospitalizations during the pandemic, *P* = 0.52. We found 131 infections in the pre-pandemic period and 75 infections during the pandemic. The percentage of nosocomial infections decreased in the pandemic period (25.3% vs. 37.4% in the pre-pandemic period, *P* = 0.06). We found a non-significant trend to a higher incidence of infections by multi-drug resistant organisms (MDRO) in the pandemic period than in the pre-pandemic period (6.5% vs. 4%). The incidence of infections was similar in both periods. However, during the pandemic, we observed a trend to a lower incidence of nosocomial infections with a higher incidence of MDRO infections.

## Introduction

Due to the widespread use of antibiotics in recent years, antimicrobial resistance has become a major public health threat to populations all over the world. Patients with cirrhosis are particularly predisposed to bacterial infections because of alterations in the immune system and invasive procedures. Such infections are a major cause of complications, acute-on-chronic liver failure (ACLF), and high short-term mortality^1–4^.

Patients with cirrhosis are highly susceptible to infections driven by multi-drug resistant organisms (MDRO) as they pose multiple risk factors for developing antibiotic resistance, such as repeated health care-exposure and need for frequent treatment with antibiotics and invasive procedures. Infections by MDRO have become a major challenge in the management of patients with cirrhosis as they are associated with poor outcomes^5–7^.

In late 2019, the outbreak of coronavirus disease 2019 (COVID-19), caused by severe acute respiratory syndrome coronavirus 2 (SARS-CoV-2), rapidly developed into a global health emergency. In order to control COVID-19, major changes -including reorganization of healthcare, physical distancing, use of personal protective equipment, emphasized hand hygiene, restricted mobility and telemedicine- were implemented worldwide. Some of these measures could have influenced the type and incidence of bacterial infections, especially those caused by MDRO^8^. Although a decrease in MDRO infections could be expected, reports addressing this issue show contradictory results^9–11^.

The aim of this study was to assess the impact of the COVID-19 pandemic on the type of infections, the incidence of nosocomial infections, and the incidence of MDRO in hospitalized patients with cirrhosis during the pandemic.

## Methods

### Patients

We conducted a retrospective medical record review to study the infections occurring in patients ≥ 18 years old who were hospitalized for acute decompensation of cirrhosis in the hepatology department at a tertiary academic hospital between 11th March 2020 and 10th March 2021 (pandemic group). We compared these infections with those occurring in similar patients admitted to the centre in the previous year, from 11th March 2019 to 10th March 2020 (pre-pandemic group). Patients were excluded from the study if they: (1) had been electively admitted for a given procedure, (2) were liver transplant recipients, or (3) were positive for SARS-CoV-2 PCR at admission (as they were hospitalized in the specific COVID-19 hospitalization areas). Because infections were the target of the study, the analysis was based on episodes of infection, not on the admissions.

We recorded relevant baseline, demographic, clinical and analytical data.

Diagnosis of cirrhosis was established by histology or by clinical, analytical, and ultrasonographic findings. Acute decompensation of cirrhosis was defined as development of ascites, gastrointestinal (GI) bleeding, hepatic encephalopathy (HE), or bacterial infection according to previously defined criteria^12^. We also included those admissions due to jaundice in the context of alcoholic hepatitis, and acute kidney injury (AKI). The severity of liver disease was assessed using the Child–Pugh score and the model for end-stage liver disease (MELD) score.

### Assessment of infections

Episodes of infection were systematically searched and identified by reviewing all the electronic medical records of the admissions. Two data abstractors were trained to fill the data abstraction form once the study variables were defined.

Bacterial, viral and fungal infections were diagnosed according to conventional criteria. Spontaneous bacterial peritonitis and spontaneous bacteremia were defined according to EASL and AASLD guidelines, while for the rest of infections the CDC/NHSN surveillance definitions and criteria were used^13–16^. Infections were classified as community-acquired, healthcare-associated (HCA), and nosocomial infections. Infections diagnosed at admission or within 48 h of hospitalization were classified as community-acquired, except for those in patients with a previous contact with a healthcare environment (hospitalization or short-term admission for at least 2 days in the previous 90 days, residence in a nursing home or a long-term care facility, or chronic haemodialysis) which were considered HCA infections. Infections developed after the first 48 h of hospitalization were considered nosocomial^5^.

Microbiological cultures and antibiotic susceptibility tests were performed according to standard international criteria. MDR bacteria were defined as non-susceptibility to at least 1 agent in at least 3 antimicrobial categories^14,17^.

### Follow-up and complications

Antibiotic treatment was implemented according to international guidelines and local policies^14^. Patients were followed throughout the hospitalization. During hospitalization we collected data concerning the development of ACLF (defined according to the EASL-CLIF Consortium criteria^18^), septic shock, the need for mechanical ventilation, and mortality.

### Statistical analysis

We compared categorical data using the chi-square test and Fisher's exact test. Continuous variables were compared using the Student *t* test if a normal distribution could be assumed, and the Mann–Whitney U test if not. We assessed normality of the continuous variables using the Kolmogorov–Smirnov test. All P-values were two-tailed and were considered significant at *P* < 0.05. Calculations were performed using SPSS 25.0 (SPSS, Chicago, IL) statistical package.

### Ethical approval

The present study, as an observational and retrospective study, was approved by the local clinical research ethics committee (Comitè d’Ètica d’Investigació amb Medicaments-CEIm) and was conducted according to the principles of the 1975 Declaration of Helsinki. Waiver of obtaining informed consent from the included patients was provided by Comitè d’Ètica d’Investigació amb Medicaments-CEIm due to the retrospective nature of the study, in a pandemic situation. Moreover, a high number of patients died before the study was performed and patients' data were anonymised.

## RESULTS

### Patients, admissions and infections

**Figure 1:**
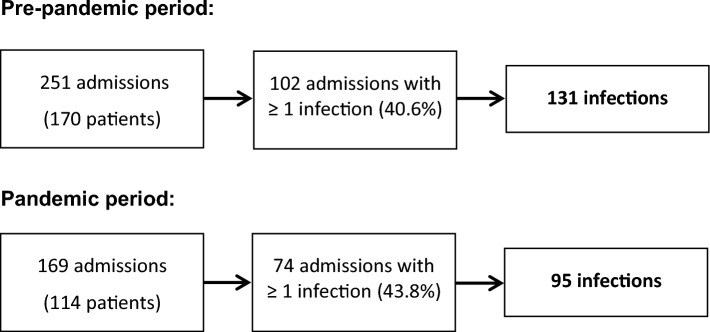
Flow chart of the study.

During the pre-pandemic period, 170 patients were admitted for a total of 251 hospitalizations due to acute decompensation, and 114 patients for 169 hospitalizations during the pandemic period (Fig. 1). The admission rate was lower in the pandemic group [23.3 (6) vs. 15.8 (4.5) admissions per month, *P* = 0.002].

One or more infections were present at admission and/or developed during hospitalization in 102/251 hospitalizations (40.6%) during the pre-pandemic period and 74/169 hospitalizations (43.8%) during the pandemic period (*P* = 0.52). The main causes for admission in hospitalizations with one or more infections were (multiple causes of admission were identified in some cases): ascites (63.7%% in the pre-pandemic period and 63.5% in the pandemic), HE (26.5% and 32.4%), portal hypertension related GI bleeding (3.9% and 6.8%), and infection (40.2% and 50%) (P NS). Other causes of admission were AKI (28.4% and 20.3%), jaundice (2% and 2.7%) and non-portal hypertension related GI bleeding (3.9% and 1.6%) (P NS).

Regarding the number of infections per admission, in the pre-pandemic group we detected one infection per admission in 78 admissions, two infections per admission in 21, and three or more infections in 3. In the pandemic group, we detected one infection per admission in 61 admissions, two infections per admission in 8, and three or more infections in 5. In the admissions with multiple infections, at least one infection was diagnosed at admission in 16/24 admissions during the pre-pandemic period and in 10/13 admissions during the pandemic.

Overall, we identified a total of 131 episodes of infection in 102 admissions in the pre-pandemic group and 95 episodes of infection in 74 admissions in the pandemic group. These infections were the target of the present study.

### Characteristics of infected patients

Demographic and clinical characteristics of infected patients are summarized in Table 1. The mean age was similar in both groups and most patients were men although there was a significant increase in the percentage of women during the pandemic. There were no differences between groups regarding antibiotic prophylaxis or use of non-selective beta-blockers or proton-pump inhibitors. The most common etiology of cirrhosis was alcohol in both periods although there was a statistically significant increase in this etiology during the pandemic.

**Table 1:** Clinical and analytical baseline characteristics of 131 infections in 102 admissions during the pre-pandemic period and 95 infections in 74 admissions during the pandemic period.

	Pre-pandemic n = 131	Pandemic n = 95	*P* value
Age (y)	64 ± 14	66 ± 10	0.52
Gender M/F	88 (67.2%)/43 (32.8%)	50 (52.6%)/45 (47.4%)	**0.03**
Etiology, n (%)			
Alcohol	58 (44.3%)	57 (60%)	**0.01**
Virus	20 (15.3%)	8 (8.4%)	0.18
Alcohol + virus	19 (14.5%)	14 (14.8%)	0.88
MAFLD	19 (14.5%)	8 (8.4%)	0.23
Others	15 (11.5%)	8 (8.4%)	0.60
Type 2 diabetes	56 (42.7%)	40 (42.1%)	0.92
Antibiotic prophylaxis	39 (29.8%)	19 (20%)	0.10
Beta-blockers prophylaxis	10 (53.4%)	45 (47.4%)	0.37
PPI treatment	72 (55%)	57 (60%)	0.45
Hepatocellular carcinoma	34 (26%)	16 (16.8%)	0.10
Child–Pugh pre-admission	7 ± 2	7 ± 2	0.65
MELD pre-admission	9 ± 4	12 ± 5	** < 0.001**
Child–Pugh at infection	9 ± 2	9 ± 2	0.30
MELD at infection	17 ± 7	17 ± 5	0.55
Sodium (mmol/L)	134 ± 6.1	136 ± 6.1	**0.03**
Creatinine (µmol/L)	120 ± 73.9	121 ± 95.2	0.28
Bilirubin (µmol/L)	74 ± 116	58 ± 55	0.93
Albumin (g/L)	27.7 ± 5.8	28.2 ± 5.5	0.42
INR	1.50 ± 0.5	1.6 ± 0.5	0.90
C-reactive protein (mg/L)	62.2 ± 53.4	54.4 ± 59.1	0.06
WBC (× 10^9/L)	9.67 ± 6.4	7.23 ± 4.2	**0.01**

Results are expressed as mean ± standard deviation or n (percentage).

*MAFLD: metabolic associated fatty liver disease*; *PPI*: proton pump inhibitors; *MELD:* model for end-stage liver disease*; WBC*: white blood cells.

Statistically significant values are in bold.

Prior to admission, pandemic patients had more impaired liver function according to MELD [12 (5) vs. 9 (3.7), *P* < 0.001] but Child–Pugh score was similar. At diagnosis of the infection, we found no differences in MELD [17 (5.4) in the pandemic group vs. 17 (6.5), *P* = 0.55] or in the Child–Pugh score [9 (2) vs. 9 (2), *P* = 0.29].

### Type of infections

**Figure 2:**
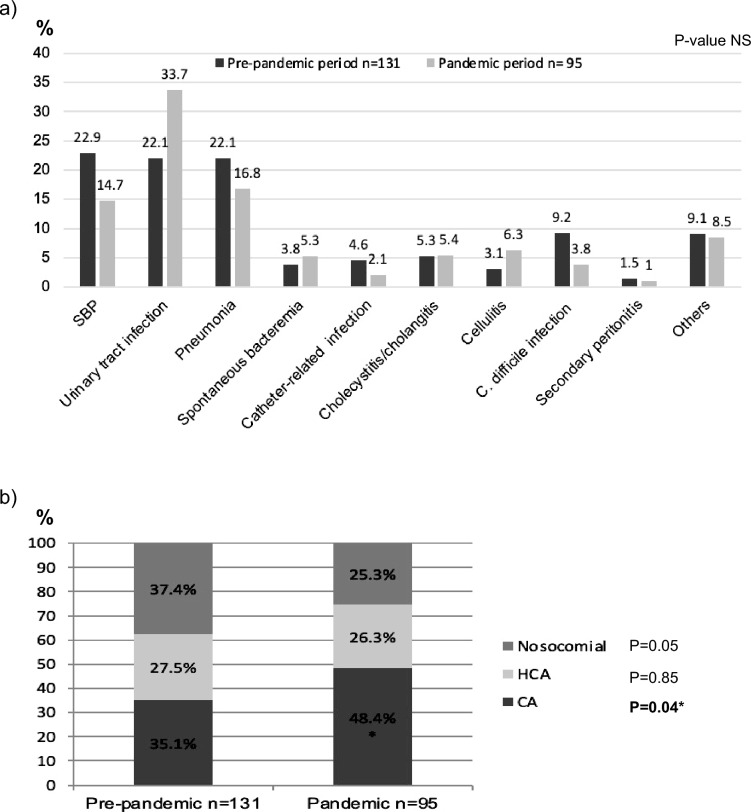
(**a**) Type of infections. (**b**) Site of acquisition of infections. *SBP*: spontaneous bacterial peritonitis; *NS*: not significant; *HCA*: healthcare-associated; *CA*: community-acquired.

The main characteristics of infections are shown in Fig. 2a. The most common infections were spontaneous bacterial peritonitis (SBP), urinary tract infection, and pneumonia. During the pandemic period we observed a non-significant reduction in SBP and pneumonia (14.7% vs. 22.9% and 16.8% vs. 22.1% of all infections, respectively) and a non-significant increase in urinary tract infections (33.7% vs. 22.1%). We detected a non-significant decrease in *Clostridioides difficile* infections during the pandemic period (9.2% vs. 3.8%).

### Site of acquisition

In the pandemic group, the percentage of community-acquired infections increased (48.4% vs. 35.1% in the pre-pandemic period, *P* = 0.04) and the percentage of nosocomial infections decreased (25.3% vs. 37.4%, *P* = 0.06) (Fig. 2b). Considering all admissions, the incidence of nosocomial infections was lower during the pandemic but did not reach statistical significance [18/169 admissions (10.7%) vs. 35/251 admissions (13.9%), *P* = 0.32].

### Microbiological cultures and MDRO

A positive microbiological culture was found in 71/131 infections (54.2%) in the pre-pandemic group and in 59/95 infections (62.1%) in the pandemic group (*P* = 0.23). The types of microorganisms are detailed in Supplementary Table 1. Gram-negative bacteria were the most commonly isolated bacteria in both groups (45.1% in the pre-pandemic group vs. 50.9% in the pandemic group). Gram-positive bacteria, anaerobic bacteria, and mixed cultures were also frequent in both periods (28.2% vs. 20.4%, 11.3% vs. 6.8%, and 11.3% vs. 10.1%, respectively) followed by viral and fungal infections (2.8% vs. 6.8% and 1.4% vs. 5.1%, respectively) without statistical differences. Only 3 patients in the pandemic group had SARS-CoV-2 acquired during hospitalization.

**Figure 3:**
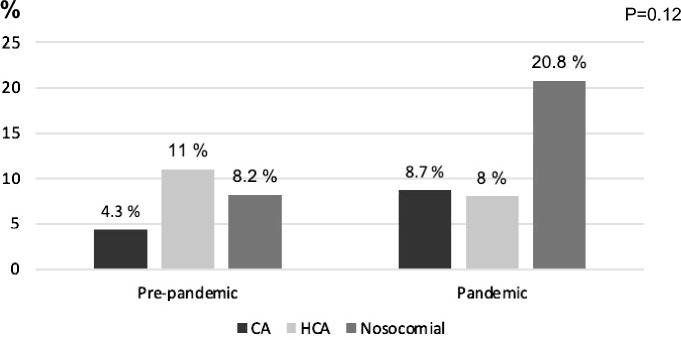
Site of acquisition of multi-drug resistant bacteria among the total of infections. *HCA*: healthcare-associated; *CA*: community-acquired.

We found a non-significantly higher percentage of MDRO among the total infections in the pandemic period than in the pre-pandemic period [11/95 (11.5%) vs. 10/131 (7.6%), *P* = 0.31]. The types of MDRO-related infections are shown in Table 2. During the pandemic we observed an increase in MDRO-related infections among the nosocomial infections (Fig. 3). Considering all admissions, we found a trend to a higher incidence of MDRO-related infections during the pandemic (11/169 admissions, 6.5%) than in the pre-pandemic period (10/251 admissions, 4%), *P* = 0.25.

**Table 2:** Multi-drug resistant bacteria isolated from 131 episodes of infection during the pre-pandemic period and 95 episodes of infection during the pandemic period.

	Pre-pandemic n = 10	Pandemicn = 11
***E. coli*** **ESBL**		
Urinary tract infection	3	7
SBP	2	–
Spontaneous bacteremia	1	–
***K. pneumoniae*** **ESBL**		
Urinary tract infection	2	1
Spontaneous bacteremia	–	1
***P. aeruginosa***		
Urinary tract infection	–	–
Pneumonia	2	1
**MRSA**		
Urinary tract infection	–	1

*SBP:* spontaneous bacterial peritonitis*; ESBL*: extended-spectrum beta-lactamase; *MRSA*: methicillin-resistant *Staphylococcus aureus.*

### Complications during admission

There were no statistical differences between the two groups regarding the incidence of ACLF (15.3% during the pre-pandemic period vs. 12.6% during the pandemic period, *P* = 0.57), septic shock (13.7% vs. 9.5%, *P* = 0.33), need for mechanical ventilation related to the infection (2.3% vs. 5.3%, *P* = 0.28) and 30-day mortality (16% vs. 11.6%, *P* = 0.34).

## Discussion

To our knowledge, this is the first comprehensive study analyzing the impact of the COVID-19 pandemic on the incidence and type of infections in patients with cirrhosis hospitalized in a non-intensive care unit setting. The main finding of this study was the similar incidence of infections in both periods (pre-pandemic vs pandemic) although we found a trend to a lower incidence of nosocomial infections and to a higher incidence of MDRO infections during the pandemic.

During the COVID-19 pandemic, strict measures were implemented to prevent the spread of SARS-CoV-2. These measures, such as greater awareness of hand hygiene, are also recommended to prevent infections in general and MDRO infections specifically^19^. We could therefore have expected a decrease in the incidence of all types of non-COVID-19 infections during the pandemic, as has been reported in the general population in outpatient medical care in Germany^20^ and in hospitalizations in the US^21^.

In the present study in hospitalized patients with cirrhosis, the incidence of all infections was similar in the pandemic to that in the pre-pandemic period, but there was a trend to a decreased incidence of nosocomial infections during the pandemic. This finding is in agreement with a report from the US that evaluated nosocomial infections in cirrhotic patients admitted to the intensive care unit^22^, as well as a report from China that evaluated nosocomial infections in a neurosurgery unit^23^, and could be attributed to the measures implemented in hospitals to fight the pandemic. However, during the first and second year of the COVID-19 pandemic, the National Healthcare Safety Network in the US described an increase in HCA infections and a high incidence in device-related infections (bloodstream, urinary tract and ventilator-associated infections)^24,25^. One possible explanation for these findings is that, in parallel to practices intended to prevent and control the spread of SARS-CoV-2, usual medical care was compromised due to the overall pressure on health systems: higher patient volumes and severity as well as shortages of staff and supplies. Focusing resources on SARS-CoV-2 may have reduced the attention given to traditional programs for prevention of nosocomial infections, such as venous and urinary catheter care, leading to an increase in nosocomial infections^26,27^.

Interestingly, as other authors have observed, we too found a (non-significant) increase in urinary tract infections during the pandemic^22,23^. This increase could be related to deficient urinary catheter care due to the pressure on health-care systems. We also found a non-significant decrease in SBP and lower respiratory tract infections. The latter might be explained by the measures taken to reduce contact transmission and aerosol spread^23^. The low rate of COVID-19 infections in our study during the pandemic can be explained by the fact that all patients diagnosed with COVID-19 were admitted to specific COVID-19 hospitalization areas. The three patients with nosocomial-acquired COVID-19 identified during the pandemic period were diagnosed during hospitalization in the hepatology department.

Like in other studies, we also found infection rates of *Clostridioides difficile* decreased during the COVID-19 pandemic^24,25,27,28^. This finding is most likely linked to the general preventive measures undertaken during the pandemic—measures beyond the use of alcohol-based hand sanitizer to which the *Clostridioides difficile* spores are resistant.

Regarding MDRO infections, it is of note that although their incidence remained low (4% in the pre-pandemic and 6.5% in the pandemic group), it increased both in the community-acquired infections and the nosocomial infections during the pandemic period, as reported by Jeon et al in hospitalized patients in Korea^11^. In contrast, Park et al found that the rate of infections caused by MRSA and ESBL producers was significantly lower in patients with cirrhosis admitted to the intensive care unit during the pandemic. Bentivegna et al also describe a decrease in MDRO among the total numbers of infections in the general population in Rome during the pandemic^10^. Other authors in hospitals in Taiwan and the US have also described a reduction of MDRO among nosocomial infections during this period^8,9^. These contradictory findings between countries could be related to differences in antibiotic policies and/or in infection prevention measures.

We consider the low incidence of MDRO in our population in both periods is a relevant finding. Probably, the reason is that our study was performed in the hepatology unit at a hospital without liver transplant, while most of the previous studies evaluating infections by MDRO in cirrhosis were performed in an intensive-care setting and in hospitals with liver transplant program^3,5,6^. Therefore, it is important to emphasize the need for monitoring antimicrobial resistance at each centre to adapt the empiric antibiotic therapy. This would contribute to a more rational use of antibiotics in order to prevent further progression of antibiotic resistance.

In our study, the admission rate was statistically lower in the pandemic group. One hypothesis might be that patients chose to stay at home rather than seek medical care due to fear of exposing themselves to SARS-CoV-2 and/or to reduce the pressure on health care system. This hypothesis is further supported by the finding that liver function was more severely impaired in hospitalized patients during the pandemic. A possible reason for this could be that in view of the pandemic only the most seriously ill sought hospital care.

While some studies also report a decrease in the overall hospital admission rate for non-covid-19 conditions during the pandemic^29^, still others found the admission rate for alcohol-related liver events increased^30,31^. In our study, we observed a significant increase in the alcoholic etiology of cirrhosis during the pandemic. These data are in agreement with the increase in alcohol abuse during this period^32^. We observed an increase in the number of women hospitalized during the pandemic, likely related to the increase in the rate of alcohol consumption reported among women at this time and higher susceptibility to the effects of alcohol^32^.

The main limitation of this study is its retrospective design. Data reliability is not the same as in a prospective design in a non-pandemic situation. Second, we focused only on the two periods we considered could best show the impact of the pandemic on infections: the first year of the pandemic and the previous year. This provided a relatively low sample size that could have contributed to the lack of a statistical significance in some differences observed between the two periods. Finally, we did not include patients with cirrhosis admitted for COVID-19, so we do not have data about their eventual episodes of infection.

To conclude, we observed a trend towards a lower incidence of nosocomial infections and a higher incidence of MDRO infections during the pandemic as compared to the pre-pandemic period. Such findings indicate that even in a crisis like a pandemic, standard infection prevention practices must be maintained in order to avoid the increase of nosocomial infections and the spread of MDRO infections.

### Supplementary Information

Supplementary Information.

## Data Availability

The datasets used and/or analysed during the current study are available from the corresponding author on reasonable request.

## Abbreviations

ACLF: Acute-on-chronic liver failure
MDRO: Multi-drug resistant organisms
SARS-CoV-2: Severe acute respiratory syndrome coronavirus 2
MELD: Model of end-stage liver disease
HCA: Healthcare-associated
GI: Gastrointestinal
HE: Hepatic encephalopathy
AKI: Acute kidney injury
SBP: Spontaneous bacterial peritonitis
