# Supplementary material for: Impact of the COVID-19 pandemic on the incidence and type of infections in hospitalized patients with cirrhosis: a retrospective study
Source: Sci Rep. 2024 Feb 1;14:2718. doi: 10.1038/s41598-024-52452-2 (PMC10834517; doi:10.1038/s41598-024-52452-2)
Supplement: Supplementary file 1 — Supplementary Information. [file 41598_2024_52452_MOESM1_ESM.docx]

**Impact of the COVID-19 pandemic on the incidence and type of infections in hospitalized patients with cirrhosis: a retrospective study**

**Authors: Berta Cuyàs^1^; Anna Huerta^1^; Maria Poca^1,2^; Edilmar Alvarado-Tapias^1,2^; Anna Brujats^1^; Eva Román^1,2,3^; Carlos Guarner^1,2^; Àngels Escorsell^1,2^; German Soriano^1,2^**

**SUPPLEMENTARY TABLE**

Supplementary table 1. Microorganisms isolated in positive culture-infections.

|  | **Pre-pandemic**  **n=71** | **Pandemic**  **n=59** | **P** |
| --- | --- | --- | --- |
| **Gram-negative bacteria**   - *E. coli* - *K. pneumoniae* - *P. aeruginosa* - *S. marcescens* - *R. ornithinolytica* - *Acinetobacter* | **32 (45.1%)**  22 (31%)  6 (8.5%)  3 (4.2%)  1 (1.4%)  -  - | **30 (50.9%)**  23 (39%)  4 (6.8%)  1 (1.7%)  -  1 (1.7%)  1 (1.7%) | 0.51 |
| **Gram-positive bacteria**   - *S. epidermidis* - *S. aureus* - *E. faecium* - *E. faecalis* - *S. capitis* - *S. gordonii* - *S. hominis* - *S. oralis* - *S. salivarius* - *S. gallyticus* - *S. agalactiae* | **20 (28.2%)**  5 (7.1%)  4 (5.6%)  4 (5.6%)  2 (2.8%)  1 (1.4%)  1 (1.4%)  1 (1.4%)  1 (1.4%)  1 (1.4%)  -  - | **12 (20.4%)**  2 (3.4%)  2 (3.4%)  3 (5.1%)  3 (5.1%)  -  -  -  -  -  1 (1.7%)  1 (1.7%) | 0.30 |
| **Anaerobic bacteria**   - *C. difficile* - *P. denticola* | **8 (11.3%)**  7 (9.9%)  1 (1.4%) | **4 (6.8%)**  4 (6.8%)  0 | 0.54 |
| **Mixed** | **8 (11.3%)** | **6 (10.1%)** | 0.84 |
| **Virus**   - Cytomegalovirus - Influenza A - SARS-CoV-2 - Herpes simplex virus type 1 | **2 (2.8%)**  1 (1.4%)  1 (1.4%)  -  - | **4 (6.8%)**  -  -  3 (5.1%)  1 (1.7%) | 0.41 |
| **Fungi**   - *C. albicans* - *C. tropicalis* | **1 (1.4%)**  1 (1.4%)  - | **3 (5.1%)**  2 (3.4%)  1 (1.7%) | 0.33 |
